# Association between obesity and cardiac conduction defects

**DOI:** 10.3389/fcvm.2025.1476935

**Published:** 2025-07-09

**Authors:** Mohamed A. Mostafa, Jeff A. Kingsley, Elsayed Z. Soliman, Prashant D. Bhave

**Affiliations:** Department of Cardiovascular medicine, Wake Forest School of Medicine, Winston-Salem, NC, United States

**Keywords:** BMI, obesity, cardiac conduction defects, heart block, UK biobank

## Abstract

**Background:**

Literature on the association between high body mass index (BMI) and cardiac conduction defects (CCD) is scarce.

**Methods:**

The cross-sectional association between obesity and CCD was examined in 455,790 participants (56.1 years; 55.9% females) from the United Kingdom (UK) Biobank. CCD was defined by ICD codes as the presence of either atrioventricular block (AVB) or intraventricular block (IVB). Multivariable logistic regression models were used to assess the association between different levels of BMI and CCD.

**Results:**

About 2.7% (*n* = 12,169) of the participants exhibited CCD. Each 1-SD increase in BMI (4.68 kg/m^2^) was associated with increased odds of CCD (OR (95% CI): 1.03 (1.01, 1.06). In subgroup analysis, this association was stronger in older participants (>65 vs. <65 years), men than women, and participants with diabetes (interaction *p*-value < 0.05 for all). In a stratified analysis by CCD subtypes, each 1-SD of BMI was associated with increased odds of AVB, but not IVB [OR (95% CI): 1.04 (1.01, 1.07), 0.97 (0.89, 1.05), respectively]. Compared to normal BMI (25–29.9 Kg/m^2^), participants with marked obesity, defined as BMI >40 Kg/m^2^, had 20% increased odds of CCD (OR (95% CI): 1.20 (1.04, 1.39). No significant association was observed with BMI between 30 and 39.9 Kg/m^2^.

**Conclusions:**

Higher BMI levels are associated with an increased risk of CCD, which is probably triggered by AVB, and the association is stronger in men, the elderly, and those with diabetes; further research is needed to examine whether weight management in obesity will be accompanied by a reduction in the risk of CCD.

## Introduction

Cardiac conduction defects (CCD) is a prevalent condition characterized by disruptions in the heart's normal electrical depolarization. It encompasses a spectrum of symptoms given its pathophysiological nature, affecting various levels of the cardiac conduction pathways. These symptoms range from benign electrocardiographic findings to potentially life-threatening heart rhythm disturbances or heart block (1, 2). CCD is an established predictor of heart failure and cardiovascular mortality (3). The usual course of treatment for symptomatic end-stage CCD is cardiac pacing. While pacemakers provide a solution for CCD, this treatment comes with its own set of challenges, such as financial burdens, infection risk, the need for serial generator changes, and potential adverse health effects associated with chronic pacing itself (4). In 2015 it was estimated that 12% of adults were obese worldwide, with an attributable 3 million deaths every year (5). More than two-thirds of the deaths associated with a high BMI are caused by CVD (5). Only few studies have examined the effects of lifestyle behaviors on bradyarrhythmia and CCD (6, 7). With the clear need for strategies to mitigate the burden of CCD, identifying modifiable risk factors is essential. Given the scant evidence, we aimed to assess association of high BMI with the risk of CCD and its subtypes in the general population using UK Biobank cohort.

## Materials and methods

**Figure 1:**
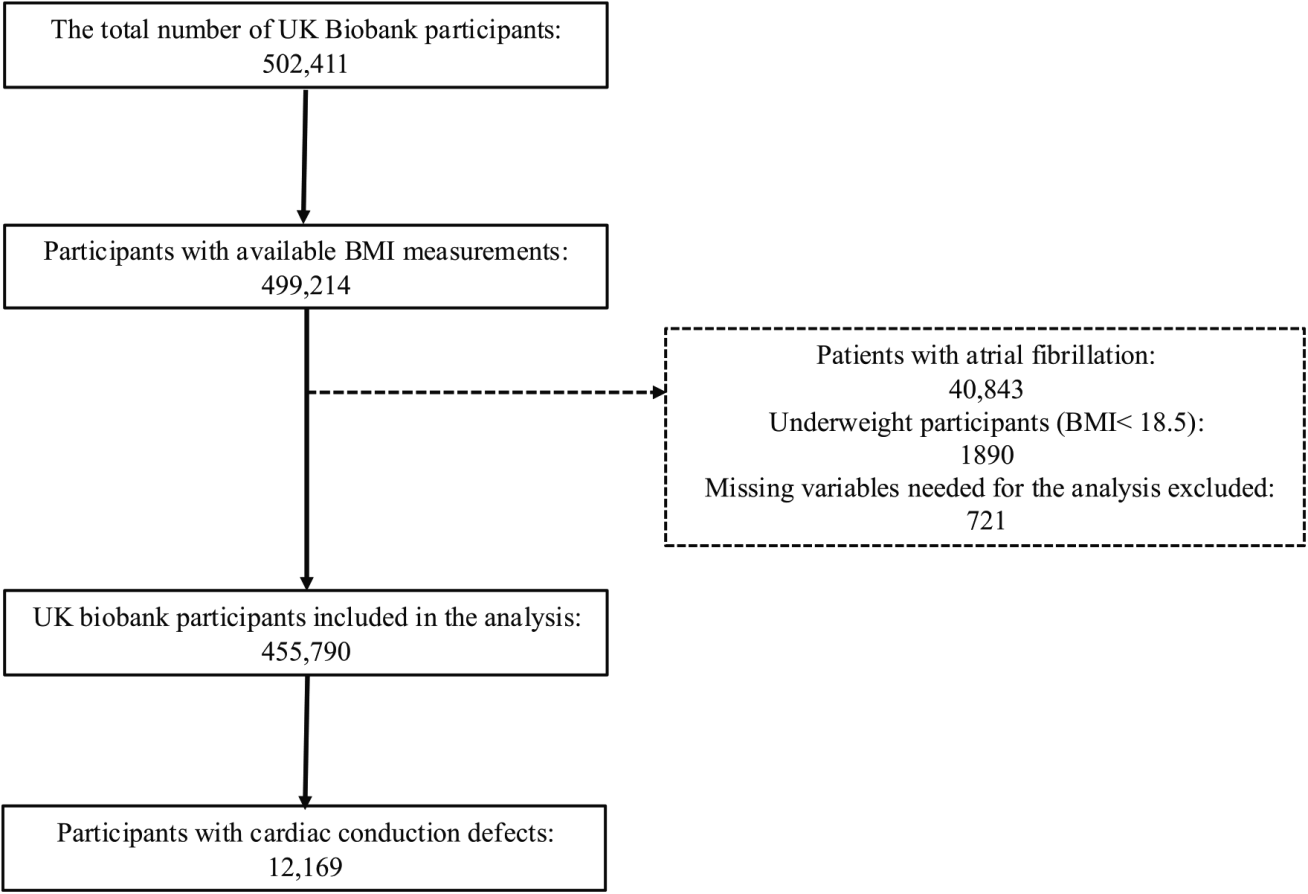
Flow chart for study population.

The UK Biobank is a prospective cohort study that enrolled over half a million participants aged 40–69 years between 2006 and 2010 from across the UK. Participants attended one of the 22 assessment centers located across England, Scotland, and Wales, where they completed touchscreen and nurse-led questionnaires, underwent physical measurements, and provided biological samples. Information on sociodemographic factors, habitual diet, lifestyle, medical history, and medication usage was collected through touchscreen questionnaires at recruitment (8, 9). The UK Biobank study received approval from the National Information Governance Board for Health and Social Care in England and Wales, the Community Health Index Advisory Group in Scotland, and the Northwest Multicenter Research Ethics Committee. All participants provided written informed consent, and the study was approved by the National Research Ethics Service. Those who were underweight (BMI < 18.5) or with missing variables needed for the analysis were excluded (<2% of the total cohort). Additionally, we excluded those with atrial fibrillation from our sample as shown in the flow chart in Figure 1 The final analysis included 455,790 participants.

Body mass index (BMI), as a primary exposure, was measured as part of the baseline during the initial enrollment. BMI was calculated as weight in kilograms divided by height in meters squared. Body weight was measured to the nearest 0.1 kg using a Tanita BC418MA body composition analyzer (Tanita, Tokyo, Japan). Height was measured using a Seca 202 height measure with the head positioned in the Frankfort plane. Obesity is defined as a BMI equal to or exceeding 30 kg/m^2^. Furthermore, BMI is subcategorized into the following groups: normal weight: BMI 18.5–24.9 kg/m^2^, overweight: BMI 25.0–29.9 kg/m^2^, obese: BMI 30.0–39.9 kg/m^2^, severe obesity: BMI ≥ 40 kg/m^2^.

The primary outcome for this analysis is composite CCD, which is defined as first, second or third-degree atrioventricular block (AVB), complete or incomplete right bundle branch block (RBBB), complete or incomplete left bundle branch block (LBBB), left anterior fascicular block (LAFB), left posterior fascicular block (LPFB), bifascicular or trifasciuclar block or nonspecific intraventricular conduction delay. Cardiac conduction defects were subdivided into two groups: atrioventricular block (AVB) and intraventricular block (IVB). AVB included participants with first-, second-, or third-degree AV block. IVB defined as those with RBBB, LBBB, LPFB, LAFB, bifascicular block, trifascicular block or nonspecific intraventricular block. Similar classification has been utilized in prior literature (6, 7, 10, 11). Prevalent CCD was extracted from the first occurrence of health outcomes defined by the 10th three-character International Statistical Classification of Diseases (ICD-10).

Our analysis was adjusted for sociodemographic, behavioral, and lifestyle factors, common CVD risk factors and comorbidities. Information on participants' age, sex, ethnicity, educational levels, smoking status, alcohol intake frequency were self-reported based on validated questionnaires administered at baseline. Physical activity was determined using adapted questions from the validated short International Physical Activity Questionnaire. Social status was determined using the Townsend deprivation index with higher scores indicating higher levels of socioeconomic deprivation (12). Hypertension was defined as self-reported use of antihypertensive medication and/or systolic blood pressure ≥130 mmHg and/or diastolic blood pressure ≥80 mmHg, measured using the Omron HEM-7015IT digital blood pressure monitor by averaging two automated measures taken one minute apart. Prevalent comorbidities were determined based on self-reported physician diagnoses at baseline, along with relevant medication use or biomarker thresholds when applicable. Diabetes was defined by a self-reported diagnosis, use of glucose-lowering medications, or an HbA1c level of ≥6.5% at baseline. Coronary artery disease included a history of myocardial infarction, coronary angioplasty (with or without stenting), or coronary artery bypass grafting. Heart failure encompassed diagnoses of heart failure, cardiomyopathy, pulmonary edema, or hypertrophic cardiomyopathy. Valvular heart disease was defined by the presence of aortic or mitral stenosis or regurgitation, mitral valve prolapse, or any history of valve repair or replacement. Dyslipidemia was defined by self-reported use of lipid-lowering medications or elevated total cholesterol: >=6.2 mmol/L, high-density lipoprotein (HDL) cholesterol: <1.0 mmol/L, triglycerides: >=2.3 mmol/L, low-Density Lipoprotein (LDL) Cholesterol: >=4.1 mmol/L. Demographics and clinical characteristics of the participants were compared across BMI groups using student ANOVA for continuous variables and Chi-square for categorical variables. Given the large sample size, we reported effect sizes as eta-squared (η²) for continuous variables and Cramér's V for categorical variables. Odds ratios (OR) and 95% confidence intervals (CI) for the risk of CCD and its subtypes were estimated using multivariable logistic regression models across BMI groups, with the normal weight group as a reference. To examine the association between BMI and CCD, three multivariable-adjusted models were constructed. Model 1 adjusted for demographics (age, sex, and race) and model 2 adjusted for model 1 plus education level, social status, smoking status, alcohol consumption, diabetes, hypertension, dyslipidemia, and prior CVD including coronary heart disease, heart failure, atrial fibrillation and valvular heart disease. Final model 3 accounted for physical activity in addition to model 2. Similar models were utilized to examine the odds of CCD associated with 1-SD (4.76) increase in BMI. Subgroup analysis was conducted to examine the effect modification of the association between tertiles and I-SD of BMI by age (< 65 vs. >65 years), sex (men vs. women), race (white vs. non-white), smoking status, physical activity, and diabetes. Interaction with the main effect was tested in an adjusted model similar to model 3.

All statistical analyses were performed using JMP Pro 17. A *P*-value of less than 0.05 was coincided significant.

## Results

Among 455,790 participants included, 2.7% (*n* = 12,169) had prevalent CCD. Prevalent conduction disease included 3,073 with first-degree AVB, 928 with 2nd degree AVB, 1,250 with 3rd degree AVB, 4,239 with RBBB, 3,795 with LBBB, 106 with either LAFB or LPFB, 456 with bifasicular block, 409 with trifasicular block, 101 with nonspecific IVB (3,011 participants had more than one type of CCD). Participants with more than one CCD were included in the respective CCD group if at least one type met the inclusion criteria for AVB, IVB, or CCD groups. The mean BMI of the total population was 27.3 ± 4.6, 43% were overweight and 24% were obese. Baseline characteristics of study population stratified by BMI groups are shown in (Table 1). The prevalence of CCD, AVB, and IVB increased with higher BMI levels, peaking in participants with severe obesity compared to the normal weight group (4.2% vs. 3.5% vs. 2.8% vs. 1.9%), respectively. Participants with highest levels of BMI tended to be female, white and had higher prevalence of diabetes, CVD and dyslipidemia.

**Table 1:** Baseline population characteristics.

BMI groups/number	BMI 18.5–24.9	BMI 25.0–29.9	BMI 30.0–39.9	BMI > 40	*P*-value	Effect size
	*N* = 148,616	*N* = 1,97,074	*N* = 1,01,919	*N* = 8,181		
Age, years	55.3	56.5	56.4	54.5	<0.001	0.006
Sex, males N (%)	49,783 (33.5%)	101,326 (51.4%)	47,775 (46.8%)	2,327 (28.4%)	<0.001	0.164
Race, Whites N (%)	137,430 (92.5%)	189,860 (96.3%)	92,673 (90.9%)	7,356 (89.9%)	<0.001	0.042
Education level, years	16.9	16.7	16.6	16.6	<0.001	<0.001
Social Status, Townsend Deprivation Index	−1.5	−1.4	−0.9	0	<0.001	0.010
Physical activity, MET						
Walking	1,030	1,021	1,035	1,044	0.154	0.027
Moderate	935	920	932	940	0.323	
Vigorous	681	669	684	678	0.192	
Smoking status *N* (%)						
Never	88,252 (59.4%)	106,714 (54.1%)	52,828 (51.8%)	4,467 (54.6%)	<0.001	0.047
Former	43,048 (29.0%)	69,203 (35.1%)	38,492 (37.8%)	2,897 (35.4%)	<0.001	
Current	16,755 (11.3%)	20,170 (10.2%)	9,965 (9.8%)	763 (9.3%)	<0.001	
Alcohol consumption *N* (%)						
Never	10,709 (7.2%)	14,072 (7.1%)	9,815 (9.6%)	1,297 (15.9%)	<0.001	0.080
Special occasions only	14,818 (10.0%)	20,268 (10.3%)	15,229 (14.9%)	2,110 (25.8%)	<0.001	
1 to 3 times per month	15,479 (10.4%)	20,943 (10.6%)	13,601 (13.3%)	1,423 (17.4%)	<0.001	
Once or twice per week	37,588 (25.3%)	51,926 (26.3%)	27,196 (26.7%)	1,869 (22.8%)	<0.001	
3 to 4 times per week	36,584 (24.6%)	48,137 (24.4%)	19,988 (19.6%)	891 (10.9%)	<0.001	
Daily or almost daily	33,151 (22.3%)	41,341 (21.0%)	15,820 (15.5%)	564 (6.9%)	<0.001	
Prefer not to answer	153 (0.1%)	190 (0.1%)	127 (0.1%)	15 (0.2%)	<0.001	
Diabetes, N (%)	3,971 (2.7%)	13,493 (6.8%)	17,167 (16.8%)	2,780 (34.0%)	<0.001	0.246
Hypertension, *N* (%)	25,843 (17.4%)	57,451 (29.2%)	44,097 (43.3%)	4,666 (57.0%)	<0.001	0.239
Coronary artery disease, *N*(%)	7,846 (5.3%)	17,945 (9.1%)	12,381 (12.1%)	1,045 (12.8%)	<0.001	0.111
Heart failure, *N* (%)	1,728 (1.2%)	3,622 (1.8%)	3,223 (3.2%)	456 (5.6%)	<0.001	0.092
Valvular disease, *N* (%)	3,312 (2.2%)	5,773 (2.9%)	3,710 (3.6%)	378 (4.6%)	<0.001	0.054
Dyslipidemia, *N* (%)	13,452 (9.1%)	30,142 (15.3%)	21,236 (20.8%)	1,978 (24.2%)	<0.001	0.142
Cardiac conduction defects (CCD), *N* (%)	2,761 (1.9%)	5,452 (2.8%)	3,610 (3.5%)	346 (4.2%)	<0.001	0.057
*Atrioventricular nodal block (AVB):*	1,113 (0.75%)	2,352 (1.19%)	1,626 (1.60%)	160 (1.96%)	<0.001	0.028
1st degree heart block, *N* (%)	572 (0.4%)	1,384 (0.7%)	1,017 (1.0%)	100 (1.2%)	<0.001	0.035
2nd degree heart block, *N* (%)	242 (0.2%)	417 (0.2%)	247 (0.2%)	22 (0.3%)	<0.001	0.013
3rd degree heart block, *N* (%)	299 (0.2%)	551 (0.3%)	362 (0.4%)	38 (0.5%)	<0.001	0.018
*Intraventricular block (IVB):*	2,038 (1.37%)	4,070 (2.07%)	2,746 (2.69%)	252 (3.08%)	<0.001	0.031
RBBB, *N* (%)	1,016 (0.7%)	1,848 (0.9%)	1,254 (1.2%)	121 (1.5%)	<0.001	0.032
LBBB, *N* (%)	846 (0.6%)	1,741 (0.9%)	1,115 (1.1%)	93 (1.1%)	<0.001	0.034
LAFB or LPFB, *N* (%)	22 (0.0%)	49 (0.0%)	34 (0.0%)	1 (0.0%)	0.001	0.005
Bifascicular block, *N* (%)	75 (0.1%)	201 (0.1%)	157 (0.2%)	23 (0.3%)	<0.001	0.002
Trifascicular block, *N* (%)	59 (0.0%)	182 (0.1%)	156 (0.2%)	12 (0.1%)	<0.001	0.011
Nonspecific IVB, *N* (%)	20 (0.0%)	49 (0.0%)	30 (0.0%)	2 (0.0%)	<0.001	0.006

RBBB, right bundle branch block; LBBB, left bundle branch block; LAFB, left anterior fascicular block; LPFB, left posterior fascicular block.

Effect sizes are reported as eta-squared (*η*²) for continuous variables and Cramér's V for categorical variables. Values ≥ 0.1 are considered clinically meaningful in this context.

In multivariable-adjusted logistic regression, increasing BMI levels were associated with higher odds of composite CCD. As shown in Table 2, in a model adjusted for demographics, prior CVD and its common risk factors, severe obesity was associated with 20% increased odds of CCD (OR (95% CI): 1.20 (1.04, 1.39). Similar patterns were observed for AVB and IVB, although the association with IVB did not reach statistical significance (OR (95% CI): 1.04 (1.01, 1.07), 0.97 (0.89, 1.05), respectively. In a continuous fashion, each 1-SD increase in BMI was associated with increased odds of CCD and AVB but not IVB, indicating a dose-response relationship (OR (95% CI): 1.03 (1.01, 1.06), 1.04 (1.01, 1.07), 0.97 (0.89, 1.05)), respectively. The association between BMI (modeled as 1-SD increase) and CCD was consistent across subgroups of the participants stratified by race, smoking status, and physical activity (Table 3). However, significant effect modification was observed, with the risk of CCD per 1-SD increase in BMI being more pronounced in older participants (>65 vs. <65), men compared to women, and participants with diabetes (interaction *p*-value = 0.035, <0.001, <0.001, respectively).

**Table 2:** Association of BMI and cardiac conduction defects.

CCD subtype	Obesity class	Unadjusted Model		Model 1		Model 2		Model 3	
		Odds Ratio	*p-value*	Odds Ratio	*p-value*	Odds Ratio	*p-value*	Odds Ratio	*p-value*
		(95% CI)		(95% CI)		(95% CI)		(95% CI)	
Composite CCD^a^	Normal weight	Ref.	–.	Ref.	–	Ref.	–	Ref.	–
	Overweight	1.50 (1.43, 1.57)	<0.001	1.22 (1.16, 1.28)	<0.001	1.02 (0.97, 1.08)	0.337	1.01 (0.95, 1.08)	0.588
	Obese	1.93 (1.84, 2.03)	<0.001	1.65 (1.56, 1.74)	<0.001	1.05 (1.00, 1.12)	0.093	1.03 (0.97, 1.11)	0.288
	Severe obesity	2.33 (2.08, 2.61)	<0.001	2.65 (2.36, 2.98)	<0.001	1.23 (1.08, 1.40)	<0.001	1.20 (1.04, 1.39)	0.013
	Per 1-SD BMI increase^d^	1.25 (1.23, 1.27)	<0.001	1.26 (1.24, 1.29)	<0.001	1.04 (1.02, 1.06)	0.026	1.03 (1.01, 1.06)	0.004
AVB^b^	Normal weight	Ref.	–.	Ref.	–	Ref.	–	Ref.	–
	Overweight	1.44 (1.36, 1.52)	<0.001	1.31 (1.24, 1.39)	<0.001	1.07 (0.99, 1.14)	064	1.01 (0.94, 1.08)	0.810
	Obese	1.83 (1.73, 1.94)	<0.001	1.72 (1.62, 1.83)	<0.001	1.19 (1.10, 1.28)	<0.001	1.05 (0.98, 1.13)	0.162
	Severe obesity	2.11 (1.84, 2.42)	<0.001	2.32 (2.02, 2.68)	<0.001	1.43 (1.21, 1.69)	<0.001	1.21 (1.02, 1.43)	0.029
	Per 1-SD BMI increase^d^	1.23 (1.21, 1.25)	<0.001	1.24 (1.21, 1.27)	<0.001	1.10 (1.08, 1.14)	<0.001	1.04 (1.01, 1.07)	0.001
IVB^c^	Normal weight	Ref.	–.	Ref.	–	Ref.	–	Ref.	–
	Overweight	1.44 (1.36, 1.53)	<0.001	1.20 (1.14, 1.28)	<0.001	1.01 (0.95, 1.07)	0.753	1.07 (0.88, 1.31)	0.455
	Obese	1.86 (1.75, 1.98)	<0.001	1.63 (1.54, 1.74)	<0.001	1.02 (0.95, 1.09)	0.620	0.81 (0.46, 1.43)	0.477
	Severe obesity	2.06 (1.78, 2.38)	<0.001	2.3 (2.04, 2.75)	<0.001	1.02 (0.87, 1.19)	0.799	1.09 (0.88, 1.34)	0.821
	Per 1-SD BMI increase^d^	1.23 (1.21, 1.25)	<0.001	1.24 (1.21, 1.27)	<0.001	1.01 (0.97, 1.03)	0.900	0.97 (0.89, 1.05)	0.492

Normal weight: BMI 18.5–24.9, Overweight: BMI 25.0–29.9, Obese: BMI 30.0–39.9, Severe obesity: BMI > 40.

Model 1 adjusted for demographics (age, sex, race) Model 2 adjusted for Model 1 plus education level, social status, smoking status, alcohol consumption, diabetes, hypertension, dyslipidemia, and CVD (CAD, HF, AF, Valvular disease) Model 3 adjusted for model 2 plus physical activity.

^a^ Composite CCD: Cardiac conduction defects defined as AVB plus IVB.

^b^ AVB: Atrioventricular Block (1st,2nd, and 3rd degree AV block plus Mobitz II).

^c^ IVB: Intraventricular block (RBBB, LBBB, LPFB, LAFB and nonspecific intraventricular block).

^d^ 1-SD BMI = 4.68.

**Table 3:** Association of BMI and cardiac conduction defects among sub-groups.

Variables	Per 1-SD BMI increase	
	Odds Ratio *(95% CI)*	*Interaction p-value* ^a^
Age		
<65	1.01 (0.98, 1.03)	0.041
>65	1.25 (0.99, 1.73)	
Gender		
Men	1.09 (1.06, 1.12)	<0.001
Women	0.99 (0.95, 1.02)	
Race		
Whites	1.03 (1.00–1.06)	0.216
Non-whites	1.08 (1.01–1.16)	
Diabetes		
Yes	1.08 (1.04, 1.12)	<0.001
No	0.99 (0.96, 1.02)	
Smoking status		
Former	1.00 (0.97, 1.03)	0.557
Current	1.02 (0.96, 1.08)	
Physical Activity		
Light	1.01 (0.92, 1.11)	0.476
Moderate	0.96 (0.87, 1.07)	0.303
Vigorous	0.90 (0.74, 1.10)	0.572

^a^ Model adjusted for demographics (age, sex, race), education level, social status, smoking status, alcohol consumption, diabetes, hypertension, dyslipidemia, CVD (coronary artery disease, heart failure, atrial fibrillation, valvular disease) and physical activity.

## Discussion

In this cross-sectional analysis of the UK Biobank, a large-scale population survey, we demonstrated a dose-response relationship between higher BMI levels and an increased risk of CCD and its subtypes, particularly AVB. This association was particularly notable among older male participants with prevalent diabetes. These findings underscore the significance of obesity as a potential risk factor for CCD and further validate prior studies linking obesity to heart arrhythmias, including CCD. Integrating obesity into risk stratification and considering it as a potential target for prevention plans could help alleviate the burden of CCD.

Extensive research explored the relationship between obesity and tachyarrhythmia such as AF (13). Current guidelines recommend weight reduction measures for AF patients with obesity, as weight loss has been shown to mitigate the occurrence, progression, and recurrence of AFs (14–16). Despite these recommendations, there are no established clinical guidelines for the prevention of bradyarrhythmia and CCD.

After adjusting for confounders and prior CVD, our analysis consistently shows that obesity is associated with CCD, particularly AVB. Furthermore, despite showing higher odds for the IVB subtype, this association was not statistically significant. Varying effects of BMI across CCDs subtypes have been reported. For incidence, Liu et al. found an increased risk of CCD in participants with obesity, but no associations were observed with IVB except for LAFB and iRBBB in a Chinese cohort while Frimodt-Moller et al. reported higher incidence of infra-Hisian block in a US-based cohort with obesity (6, 7). In terms of electrocardiogram markers, obesity has been linked to prolonged PR interval and increased QRS duration (17, 18).

While the cross-sectional nature of our analysis precludes establishing causality, the association and variation across CCD subtypes can be explained by multifactorial mechanisms involving blood supply, cellular properties, and the anatomical location of the conduction system.

Potential pathophysiological explanations mainly derived by the linear association between epicardial adipose tissue (EAT) and obesity (19). Research has demonstrated the arrhythmogenicity and adverse cardiac effects associated with EAT (20). While literature mainly focused on tachyarrhythmias including AF and ventricular arrhythmias, similar concepts apply to slowing conduction pathways and bradyarrhythmia (21, 22). Peptides and adipokines diffuse freely between EAT and the subepicardial myocardium serving as an epitome for fibrosis (23). Fatty infiltrates in EAT often coexist with fibrosis, creating non-conducting barriers between myocyte strands and potentially altering gap junctions responsible for electrical impulse propagation (20, 24, 25). This fibro-fatty infiltration serves as a common substrate for both ventricular and atrial arrhythmias, with differences attributed to specific cellular properties and electrical impulse propagation, possibly explaining the heterogeneity observed between atrial and ventricular conduction defects (26).

The observed predominance of the association between obesity and AVB, rather than IVB, may be explained by several anatomical, metabolic, and electrophysiological differences between the AV node and the His–Purkinje system. Structurally, the AV node is a compact region with a single arterial supply in over 90% of individuals, making it particularly susceptible to ischemia in the context of obesity-related microvascular dysfunction (27, 28). Its limited vascular reserve and single-entry anatomy increase vulnerability to fibro-fatty infiltration and hypertrophy (28, 29). In contrast, the His bundle and bundle branches are typically supported by dual blood supply and insulated by fibrous tissue, which may delay ischemic or inflammatory remodeling (30). Moreover, the AV node's calcium-dependent and decremental conduction properties render it more sensitive to autonomic imbalance, inflammation, and metabolic stress (31), whereas the sodium-channel–dependent His–Purkinje fibers may require more advanced or prolonged remodeling before dysfunction becomes clinically evident (32, 33).

Epicardial adipose tissue (EAT), which increases with BMI and preferentially deposits around atrial and AV junctional structures, further contributes to this differential vulnerability (20, 27). EAT secretes pro-inflammatory and profibrotic cytokines that may disrupt ion channel expression, gap junction integrity, and conduction velocity in adjacent tissues. Histological evidence shows increased collagen deposition and reduced connexin-43 in areas of fatty infiltration, particularly around the AV node (27, 29). These combined anatomical and molecular factors may explain why AVB manifests earlier or more prominently with obesity, while IVB may require a longer duration or greater burden of pathologic adiposity to become clinically apparent (27–33). Further research is needed to confirm these mechanisms and assess whether interventions such as weight loss or metabolic therapies can differentially affect the progression of CCD subtypes.

With advancements in weight reduction medication in recent years, it is important to recognize the associated cardiac outcomes and their potential to influence on cardiac electrophysiology either through the pharmacological priorities of these agents or the achieved weight reduction (34, 35). For instance, recently approved medications such as Semaglutide and Tirzepatide have shown potential to improve cardiac outcomes and possibly influence its remodelling through metabolic modulation and weight reduction. In addition to the significant reductions in C-reactive protein and NT-proBNP reported in the STEP-HFpEF trial with semaglutide use in patients with obesity and HFpEF, a subsequent substudy demonstrated further favorable effects. Notably, reductions were observed in left atrial volume, EAT volume, and biomarkers of fibrosis such as galectin-3 and TGF-β (36, 37). These findings support the potential role of Semaglutide in modifying arrhythmogenic substrates associated with conduction disease.

Similarly, a substudy of the SUMMIT trial demonstrated that that Tirzepatide improved diastolic function and reduced epicardial fat and left atrial size- factors that are key in arrhythmogenic substrates, including conduction disturbances particularly in patients with obesity (38, 39). These findings indicate that newer pharmacologic treatments for obesity may alter the trajectory of cardiac conduction disease and warrant further investigation in this context.

Our analysis revealed that the effects of obesity and CCD were most pronounced in older male participants with prevalent diabetes, consistent with prior literature showing higher risk among older individuals and men across CCD subtypes (40). EAT, which is more abundant in men compared to women and increases linearly with BMI, secretes inflammatory and profibrotic cytokines that promote fibro-fatty infiltration of the AV node and surrounding myocardium (19).

Diabetes alone has been linked to increased risk of CCD through systemic inflammation, fibrosis, and autonomic dysfunction. When combined with obesity and aging, these effects are amplified by microvascular and endothelial dysfunction, further impairing blood flow to conduction tissue (41, 42). The combination of greater baseline EAT volume, accelerated fibro-fatty remodeling, and diabetes-associated microvascular disease may together create a synergistic substrate for conduction block in this high-risk subgroup.

Our results should be interpreted within certain limitations. Our study utilized ICD-9 codes to define clinical diagnoses, a method widely adopted in large-scale population-based research. However, ICD-9 codes may not fully capture the clinical complexity of certain conditions, potentially leading to diagnostic misclassification. As a result, there is a risk of imprecise identification of comorbidities and confounding variables, which could affect the accuracy of adjusted effect estimates. Despite adjusting for potential confounders and CVD risk factors, residual confounding by other comorbidities including obstructive sleep apnoea remains possible. Additionally, the UK Biobank study's sample may not fully represent the general UK population of the same age, as volunteers were older, more likely to be white, had higher socioeconomic status, and fewer cardiovascular disease risk factors. Furthermore, different classifications of BMI groups exist and are influenced by race and ethnicity, which could yield slightly different results. To counteract this, our analysis was adjusted for race and ethnicity. Additionally, some baseline variables were self-reported and captured through a nurse-led questionnaire, which may introduce recall bias and subjectivity despite the standardized data collection process. Despite these limitations, our analysis has several strengths, including a large sample size, numerous cases, and adjustments for common cardiovascular risk factors. Lifestyle habits and baseline covariates were prospectively ascertained using uniform methods according to predefined protocols.

Our findings show that higher BMI levels are linked to increased odds of CCD in the general population. Given the limited treatment options available for CCD, it is crucial to identify modifiable risk factors to mitigate its health and economic burden. Incorporating obesity into risk stratification and implementing preventive measures, particularly among older individuals with prevalent diabetes, could help alleviate the burden of CCD.

## Data Availability

Publicly available datasets were analyzed in this study. This data can be found here: https://www.ukbiobank.ac.uk.
